# Genotoxicity Evaluation of an Ethanol Extract Mixture of* Astragali Radix* and* Salviae miltiorrhizae Radix*

**DOI:** 10.1155/2018/5684805

**Published:** 2018-10-09

**Authors:** Jin-Seok Lee, Jung-Hyo Cho, Dong-Soo Lee, Chang-Gue Son

**Affiliations:** ^1^Liver and Immunology Research Center, Oriental Medical Collage of Daejeon University, 22-5 Daehung-dong, Jung-gu, Daejeon 301-724, Republic of Korea; ^2^Department of Internal Medicine, Daejeon St. Mary's Hospital of Catholic University, Daejeon, Republic of Korea

## Abstract

Myelophil, a combination of* Astragali Radix *and* Salviae Radix,* is one of the most commonly used remedies for disorders of* Qi* and* blood* in traditional Chinese medicine. Based on the clinical applications of these plants, in particular to pregnant woman, this study aimed to evaluate the genotoxic potential of an ethanol extract mixture of the above two herbs, called Myelophil. Following the Organization for Economic Cooperation and Development (OECD) Guideline methods, a genotoxicity test was conducted using a bacterial reverse mutation test with* Salmonella typhimurium* (TA98, TA100, TA1535, and TA1537) and* Escherichia coli* (WP2*μ*vrA), an* in vitro* mammalian chromosome aberration test using a Chinese hamster ovary cell line (CHO-K1), and an* in vivo* mammalian erythrocyte micronucleus test using ICR mouse bone marrow. In the Ames test, for both types of mutations (base substitution and frameshift) under conditions with/without an S9 mix up to 5,000 *μ*g/plate, Myelophil did not increase the number of revertant colonies of all* S. typhimurium* strains as well as* E. coli* strain. For both short (6 h) and long tests with/without S9 mix, the chromosome aberration test did not show any significant increase in the number of structural or numerical chromosome aberrations by Myelophil. In addition, no significant change in the number of micronucleated polychromatic erythrocytes or polychromatic erythrocytes was observed in the bone marrow of an ICR mouse administered Myelophil orally at 2,000 mg/kg/day for 2 days, respectively. These results are the first to provide experimental evidence that Myelophil, an ethanol extract mixture of* Astragali Radix* and* Salviae Radix, *has no risk of genotoxicity.

## 1. Introduction

Herbal drugs have been used traditionally for various health benefits in East Asian countries, and they are now being adopted worldwide [1]. According to Global Industry Analysis, the global market for herbal medicinal products has grown continuously and was estimated at 107 billion US dollars in 2017. Herbal drugs are generally regarded as safe due to their derivation from natural resources and extensive clinical use for thousands of years [2]; however, recently many concerns have been raised regarding the adverse effects of herbal remedies and their low levels of quality control with standardization [3, 4].

In addition, the use of herbal products during pregnancy is common. A multinational study reported that 29.3% of pregnant women adopted herbal medicine [5]. The Swedish Medical Birth Register study reported that 787 of 860,215 women (0.9%) used herbal products during early pregnancy [6]. Accordingly, concerns about the potential risk for genotoxicity of herbal medicines and phytochemicals have arisen [7, 8]. The Korean Ministry of Food and Drug Safety (MFDS) has developed a guideline (No. 2015-82, 2015) that requires genotoxicity testing prior to introducing any herb-derived new drug.

Myelophil is a 30% ethanolic extract consisting of equal parts of* Astragali Radix* and* Salviae Radix* that are representative herbs used to treat problems of Qi and blood in traditional Korean medicine [9]. Myelophil is commonly used to maintain Qi-blood homeostasis and to treat fatigue-associated disorders, even in children and pregnant women [10]. There are subchronic toxicity studies available for* Astragali Radix* and Myelophil [11, 12]; however, no genotoxicity evaluation of Myelophil,* Astragali Radix,* or* Salviae Radix* has been conducted to date.

To provide scientific evidence about the genotoxic risk of Myelophil, we herein conducted three genotoxicity tests, i.e., a bacterial reverse mutation test, a mammalian chromosome aberration test, and a mammalian erythrocyte micronucleus test.

## 2. Materials and Methods

### 2.1. Preparation of Myelophil and Fingerprinting Analysis

Myelophil contains a 30% ethanolic extract of* Astragali Radix* (*Astragalus membranaceus*) and* Salviae Radix* (*Salvia miltiorrhiza*) in equal amounts. The Myelophil was manufactured by Kyung-Bang Pharmacy (Incheon, Rep. of Korea), according to the approved good manufacturing practice (GMP) guidelines of the Korean MFDS as previously reported [10]. Briefly, 100 kg of Myelophil was boiled in 1000 L of 30% ethanol for 4 h at 100°C and was then filtered using a 300-mesh filter (50 mm). Some samples were filtered through filter paper (Advantec, Toyo Roshi Kaisha, Tokyo, Japan) and lyophilized for this study. The final Myelophil product [yield 20.52% (w/w)] was stored for future use (VS No. KB-Myelo-1501).

In addition, for confirmation of the reproducibility of the Myelophil, fingerprinting analysis was conducted with six reference compounds: astragaloside IV (SMB003158) and formononetin (94334) for* Astragali Radix*, and salvianolic acid A (97599), B (SML0048), C (APC-657), D (APC-658), and rosmarinic acid (R4033) for* Salviae Radix* (Supplementary Figure 1). Each reference compound was purchased from Sigma (St. Louis, St. Louis, MO, USA) or Aktin Chemical, Inc., (Chengdu, China). Briefly, 1 mg of Myelophil and 10 *μ*g of each reference compound were prepared in 90 % methanol, and then filtrates were analysed using ultra-high-performance liquid chromatography (UHPLC, Thermo Scientific, San Jose, CA, USA) coupled with high resolution LTQ Orbitrap mass spectrometry (MS) system (Thermo Scientific Co., San Jose, CA, USA).

### 2.2. Bacterial Reverse Mutation Test (Ames Test)

The bacterial reverse mutation test (Test # GT17-195) was conducted by a good-laboratory-practice- (GLP-) proven laboratory (Korean Conformity Laboratories, Incheon, Rep. of Korea) in accordance with OECD guideline No. 471 (Adapted 21th July 1997). Four histidine-requiring Salmonella typhimurium strains, TA98, TA100, TA1535, and TA1537, and one tryptophan-requiring Escherichia coli strain, WP2*μ*vrA, were obtained from Molecular Toxicology Inc. (Boone, NC, USA). The positive controls were sodium azide (NaN3), 9-aminoacridine hydrochloride hydrate (9-AA), 2-(2-furyl)-3-(5-nitro-2-furyl) acrylamide (AF-2), and 2-aminoanthracene (2-AA), respectively. According to the results of the preliminary dose-range test, 5,000 *μ*g/plate was selected as the highest concentration followed by 1667, 556, 185, and 62 *μ*g/plate in the present study. The present study was tested using triplicates for each dose for each bacterial strain with or without the S9 mix. Briefly, we added 0.05 ml of Myelophil, distilled water (negative control) or positive control (AF-2 0.01 or 0.1 *μ*g/plate, NaN3 0.5 *μ*g/plate, 9-AA 80 *μ*g/plate or 2-AA 0.5 to 10 *μ*g/plate) to 2.0 ml of top agar (held at 45°C) along with a 0.1 ml precultured tester strain and 0.5 ml of either the S9 mix or 0.2 M phosphate buffer (pH 7.4). The mixture was vortexed then poured onto a minimal medium agar plate. After the agar overlay solidified, the plates were inverted and incubated at 37°C for 48 h. Following incubation, we manually counted the revertant colonies.

### 2.3. *In Vitro* Mammalian Chromosome Aberration Test

The chromosome aberration test (Test # GT17-196) was conducted by a GLP-proven laboratory (Korean Conformity Laboratories, Incheon, Rep. of Korea) in accordance with OECD guideline No. 473 (Adapted 29th July 2016). The Chinese hamster ovary cell line (CHO-K1) was obtained from the Korean Cell Line Bank (KCLB, Seoul, Korea) and cultured in minimum essential medium (Gibco, California, USA) supplemented with 10% foetal bovine serum (Gibco) at 37°C and 5% CO_2_. To determine the maximum concentration for the present study, the proliferation assay was conducted up to a dose of 5,000 *μ*g/mL with or without the S9 mix. Based on the results from the proliferation assay, the present study was designed to apply Myelophil to CHO-K1 cells without the S9 mix (up to 185.19 *μ*g/mL for 24 h, and 6 h followed by 18 h for recovery) or with the S9 mix (up to 555.56 *μ*g/mL for 6 h followed by 18 h for recovery), respectively. Mitomycin C (MMC, 0.04 *μ*g/mL) or cyclophosphamide monohydrate (CPA, 10 *μ*g/mL) was used as a positive control in the S9 mix absent or S9 mix present conditions, respectively. Following incubation and staining with 5% Giemsa (Merck, Germany, # HX69072204), we examined the structural and numerical chromosomal aberrations under an optical microscope.

### 2.4. *In Vivo* Mammalian Erythrocyte Micronucleus Test

The* in vivo* mammalian erythrocyte micronucleus test (Test # GT17-197) was conducted by a GLP-proven laboratory (Korean Conformity Laboratories, Incheon, Rep. of Korea) in accordance with OECD guideline No. 474 (Adopted July 21, 2016). Specific pathogen-free male (37) and female (12) ICR mice (7 weeks old, 30–32 g) were obtained from Orient Co., Ltd. (Seongnam, Korea). After one-week acclimatization, a dose-finding test was conducted using each of 12 male and female mice given Myelophil (each 3 mice for three dose groups of 500, 1000, or 2000 mg/kg bw/day, orally once a day) for 4 days. Based on no treatment-related clinical signs and no differences between males and females, Myelophil was administered once daily for two days by gavage to 25 male mice (each 5 mice for 0, 500, 1000, 2000 mg/kg and a positive control) for the micronucleus test. MMC (2 mg/kg bw/day in normal saline, only once on the final day of drug administration) was administered by intraperitoneal injection as a positive control. Mice were sacrificed by CO_2_ gas inhalation at 24 h after the last administration and their bone marrow cells were prepared as described [13]. We determined the proportion of immature erythrocytes (polychromatic erythrocytes, PCE) to total erythrocytes (immature plus mature erythrocytes, normochromatic erythrocytes, NCE) for each animal by analysing at least 500 erythrocytes. In addition, a minimum of 4,000 PCE was scored for the incidence of micronucleated polychromatic erythrocytes (MNPCE).

This study was reviewed and assessed by the Institutional Animal Care and Use Committee (IACUC) of the Korean Institute of Toxicology (IA17-00520). All animals were cared for in accordance with the principles outlined in the National Institutes of Health (NIH) Guide for the Care and Use of Laboratory Animals.

### 2.5. Statistical Analysis

No statistical analysis was conducted on the Ames test results. Instead, the presence of at least dose-dependent one strain or reproducible increased colony pattern at over a certain concentration indicated a positive result. The statistical analyses for the* in vitro* chromosome aberration test were performed as described previously [14]. The number of aberrant metaphases and the number of [polyploidy (PP, ≥37 chromosomes) + endoreduplication (ER)] were analysed by comparing them to the negative and positive controls using an *χ*2 test and Fisher's exact test.* In vivo* micronucleus results were evaluated according to the minor-modified methods by Lovell's study [15], and the statistical analysis was performed as previously described [16]. Statistical analyses were conducted using the SPSS 12.0 K program, and the differences were regarded as significant at P < 0.05.

## 3. Results

### 3.1. Bacterial Reverse Mutation Test (Ames Test)

**Figure 1:**
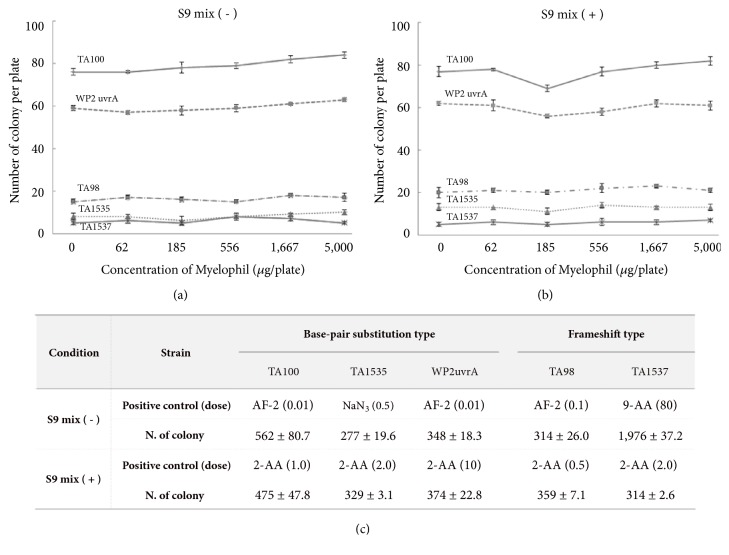
**Bacterial reverse mutation test: **five bacterial strains were treated with Myelophil in the absence (a) or presence (b) of S9 mix. The significant increase of revertant colonies by positive control verified the adequate condition of method used (c).

Myelophil did not show any cytotoxicity in* Salmonella typhimurium* (TA98, TA100, TA1535, and TA1537) or* Escherichia coli* (WP2uvrA) at up to 5,000 *μ*g/plate. The positive control compounds significantly increased the numbers of revertant colonies by more than 5 folds compared to the negative control, confirming the sensitivity of the test system (Figure 1(c)). No biologically relevant increase in revertant colonies following treatment with Myelophil at up to 5,000 *μ*g/plate was observed in any of the five tester strains in the absence or presence of S9 metabolic activation (Figures 1(a) and 1(b)).

### 3.2. Chromosome Aberration Test

Based on the cell proliferation inhibition assay, the maximum concentration of Myelophil was determined to be 185.19 *μ*g/mL and 555.56 *μ*g/mL in the absence and presence of the S9 mix, respectively. Significant increase in frequency of chromosome aberration (per 100 metaphase cells) was observed in positive control (mitomycin C and cyclophosphamide monohydrate) compared to the negative control (p < 0.01). In contrast, there is no significant increase in aberrant metaphase including structural or numerical aberrations at any dose of Myelophil with or without S9 metabolic activation (Table 1).

### 3.3. Micronucleus Test

There was no significant change in body weight between the first and final administration in the Myelophil or mitomycin C groups. The frequency of MNPCE per 4,000 PCE significantly increased in the mitomycin C group (p < 0.01); however, no dose in the Myelophil group showed a change in the MNPCE incidence compared to the negative control. In addition, there was no significant decrease in the PCE/(PCE+NCE) compared to the negative control (Table 2).

## 4. Discussion

A genotoxicity study is a key step for risk assessment during drug development for protecting human health because various genotoxic compounds can cause a DNA damage such as cross-links, adducts, and cleavage [17]. Eventually genotoxins can lead to tumor progression [18] and congenital malformations [19]. Recently, some issues regarding herb-related genotoxicity have arisen. In particular, there are concerns about the frequent exposure of pregnant women and their foetuses to phytomedicines and herbal supplements without adequate knowledge of the safety during pregnancy [8]. An analysis of 14,551 births reported an increased risk of congenital malformations of the nervous system after exposure during the first trimester of pregnancy to* Rhizoma Coptidis*, a typical medicinal herb with the property of “clearing damp-heat” [20].

In this study, we conducted a genotoxic risk assessment of Myelophil, an ethanolic extract combining* Astragali Radix* and* Salviae Radix*. We employed a bacterial reverse mutation test, a mammalian chromosome aberration test, and a mammalian erythrocyte micronucleus test. One recent review article summarized the genotoxicity of several Chinese medicinal plants, and methanol extracts of* Elephantopus Scaber *leaf exhibited the abnormalities in both mitotic index and chromosome aberrations [21]. As expected, the positive control agents significantly induced genotoxicity, which validated the tests used. However, no genotoxic positive result was observed in Myelophil treated groups compared to vehicle control (Figure 1, Tables 1 and 2). These results indicate that Myelophil does not exhibit any genotoxic risk under the experimental conditions of this study. The above tests have been used internationally as the minimal three-test battery by the International Council for Harmonisation of Technical Requirements for Pharmaceuticals for Human Use (ICH) since 1997 [22].

Myelophil has been prescribed to a wide spectrum of patients complaining of chronic fatigue and bone marrow dysfunctions in Daejeon University Hospitals since 2002.* Astragali Radix* and* Salviae Radix*, the two components of Myelophil, are very commonly used as medicinal herbs.* Astragali Radix* has pharmacological properties such as anti-inflammatory or immunomodulatory effects [23, 24], whereas* Salviae Radix* exhibited antioxidant and cardioprotective properties [25, 26], respectively. There are many active compounds, including astragaloside IV, tanshinones, and salvianolic acids, known to be present in these two herbs [27–29]. There have been general toxicological studies for* Astragali Radix*,* Salviae Radix,* and their combination conducted previously [11, 30]. However, our current study is the first genotoxic risk assessment for* Astragali Radix *and* Salviae Radix*.

The three-test battery applied in our study has limitations, especially the use of bacterial test organisms; however, its practicality is well validated. In fact, the* Rhizoma-Coptidis*-related risk of congenital malformations is supported by the genotoxicity testing of* Hwanglyeonhaedok-tang*.* Rhizoma Coptidis* is the main herb in this formula, and revertant colonies and chromosome aberrations were observed in these tests [31]. For compounds giving negative results like Myelophil, the completion of this 3-test battery usually provides a sufficient level of safety to demonstrate the absence of genotoxic activity [32].

Taken together, it was found that Myelophil is neither mutagenic in the* in vitro* systems nor clastogenic in the* in vivo* system. Based on the above results, we can conclude that Myelophil poses no genotoxic risks. Additionally, this study will provide important information for the use of* Astragali Radix* and* Salviae Radix* as medicinal resources in the future.

## Figures and Tables

**Table 1:** Chromosomal aberrations by Myelophil with and without S9 activation.

Condition	Dose (*μ*g/mL)	Chromosome aberrations (N. in 300 cells)				Aberration rate (%)
		*Break *	*Exchange*	*Numerical *	*Sum*	
0-24h S9 mix(-)	0	1	0	0	1	0.33
	20.68	2	0	0	2	0.67
	61.73	1	1	0	2	0.67
	185.19	2	0	0	2	0.67
	MC (0.04) *∗∗*	9	64	0	73	24.33
6-18h S9 mix(-)	0	1	0	0	1	0.33
	20.68	0	1	0	1	0.33
	61.73	2	1	0	3	1.00
	185.19	2	1	0	3	1.00
	MC	14	56	0	70	23.33
6-18h S9 mix(+)	0	1	1	0	2	0.67
	61.73	0	2	0	2	0.67
	185.19	1	1	0	2	0.67
	555.56	2	0	0	2	0.67
	CPA (10) *∗∗*	10	62	0	72	24.00

Structural (chromosomal break or exchange) and numerical aberration (polyploidy or endoreduplication) were counted in 300 CHO-K1 cells. MC: mitomycin C; CPA: cyclophosphamide. *∗∗*p < 0.01 *vs.* negative control.

**Table 2:** Bone marrow micronucleus assay results of male mice.

Dose (mg/kg bw/day)	Body weight (g)		*MNPCE/4000 PCEs(Mean ± SD, %)*	*PCE/(PCE+NCE)*
	day 0	day 2		
0	34.3 ± 1.4	34.2 ± 4.5	0.19 ± 0.09	0.57 ± 0.01
500	35.1 ± 1.4	35.7 ± 1.3	0.20 ± 0.07	0.56 ± 0.01
1000	35.7 ± 1.2	35.7 ± 1.3	0.20 ± 0.08	0.54 ± 0.05
2000	34.8 ± 0.7	35.7 ± 0.8	0.23 ± 0.06	0.57 ± 0.02
MC (2.0)*∗∗*	35.5 ± 1.2	36.0 ± 1.3	9.59 ± 1.74	0.41 ± 0.05

Micronucleus assay was conducted in ICR mice after oral administration for 2 days, and then the number of MNPCE (micronucleated polychromatic erythrocytes) in 4,000 PCE (polychromatic erythrocyte) was analysed. The proportion of PCE and NCE (normochromatic erythrocytes) in 500 erythrocytes was determined. MC: mitomycin C. *∗∗*p < 0.01 *vs.* negative control.

## Data Availability

The data used to support the findings of this study are available from the corresponding author upon request.

## Additional Points

This manuscript has been checked by at least two native speakers of English in scientific and technical editing service company (Nature Publishing Group language editing). For a certificate, please see languageediting.nature.com/certificate (Reference number: 19XRCXLS-413B-2D67-E849-C20F-E569).

## Supplementary Materials

Supplementary Materials For analysis, ultra-high-performance liquid chromatography (UHPLC) coupled with high resolution LTQ Orbitrap mass spectrometry (MS) system was used for fingerprinting analysis of Myelophil. The UHPLC separation was performed on an Accela UHPLC system using an Acquity BEH C18 column (1.7 *μ*m, 100 × 2.1 mm; Waters). The column was eluted at a flow rate of 0.3 mL/min using water/0.1% formic acid and acetonitrile/0.1% formic acid as mobile phases A and B, respectively, with the following gradients: 0-1 min, 10% B (isocratic); 1-10 min, 10-90% B (linear gradient); and 10-12 min, 100% B (isocratic).
