# Supplementary material for: Genotoxicity Evaluation of an Ethanol Extract Mixture of Astragali Radix and Salviae miltiorrhizae Radix
Source: Evid Based Complement Alternat Med. 2018 Oct 9;2018:5684805. doi: 10.1155/2018/5684805 (PMC6198562; doi:10.1155/2018/5684805)
Supplement: Supplementary Materials — For analysis, ultra-high-performance liquid chromatography (UHPLC) coupled with high resolution LTQ Orbitrap mass spectrometry (MS) system was used for fingerprinting analysis of Myelophil. The UHPLC separation was performed on an Accela UHPLC system using an Acquity BEH C18 column (1.7 μm, 100 × 2.1 mm; Waters). The column was eluted at a flow rate of 0.3 mL/min using water/0.1% formic acid and acetonitrile/0.1% formic acid as mobile phases A and B, respectively, with the following gradients: 0-1 min, 10% B (isocratic); 1-10 min, 10-90% B (linear gradient); and 10-12 min, 100% B (isocratic). [file 5684805.f1.docx]

**Supplementary Figure 1**


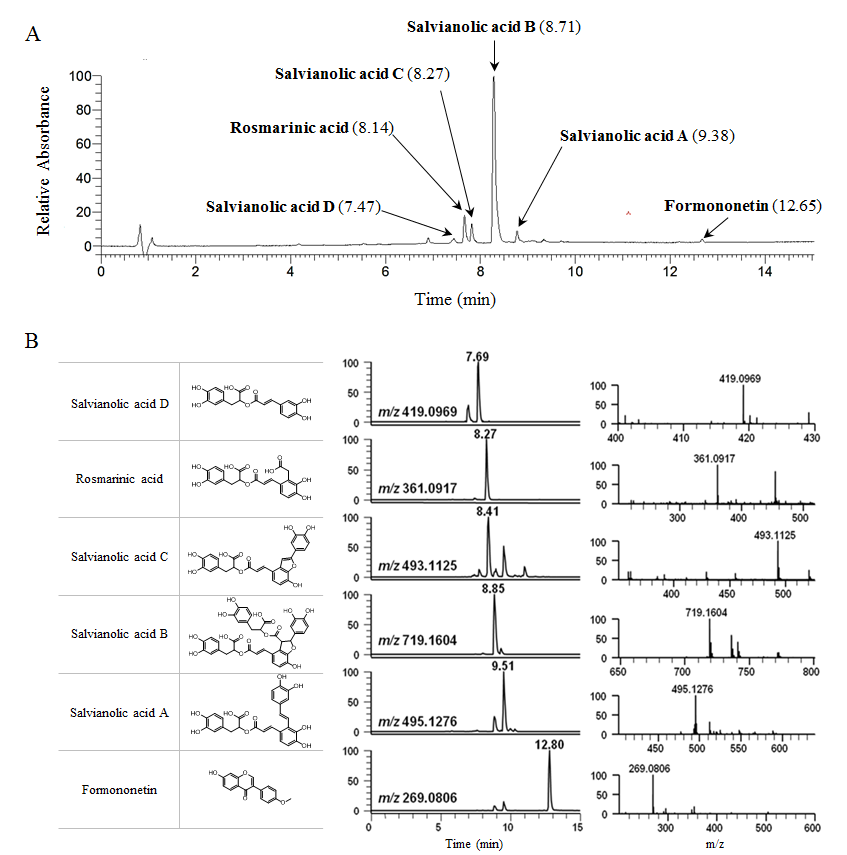


**Supplementary Figure 1.** **UHPLC and LC/MS chromatogram of Myelophil.** Myelophil and reference compounds were subjected to UHPLC analysis (A). Structures (left), mass chromatogram (middle) and high-resolution mass spectrums (right) of 6 compound of Myelophil [salvianolic acid D (m/z 419.0969, [M+H]+); rosmarinic acid (m/z 361.0917, [M+H]+); salvianolic acid C (m/z 493.1125, [M+H]+); salvianolic acid B (m/z 719.1604, [M+H]+); salvianolic acid A (m/z 495.1276, [M+H]+); formononetin (m/z 269.0906, [M+H]+)] (B).

**Supplementary Material**

For analysis, ultra-high-performance liquid chromatography (UHPLC) coupled with high resolution LTQ Orbitrap mass spectrometry (MS) system was used for fingerprinting analysis of Myelophil. The UHPLC separation was performed on an Accela UHPLC system using an Acquity BEH C18 column (1.7 μm, 100 × 2.1 mm; Waters). The column was eluted at a flow rate of 0.3 mL/min using water/0.1% formic acid and acetonitrile/0.1% formic acid as mobile phases A and B, respectively, with the following gradient: 0-1 min, 10% B (isocratic); 1-10 min, 10-90% B (linear gradient); 10-12 min, 100% B (isocratic).
